# Structure, personnel, and initiatives of antimicrobial stewardship programs in pediatric hospitals in the United States based on on-call participation: a cross-sectional survey

**DOI:** 10.1017/ash.2026.10745

**Published:** 2026-06-10

**Authors:** Gustavo Rey Alvira-Arill, Samantha Brace, Taylor Morrisette, Stephen Thacker, Aaron Hamby, Rachel Burgoon, Zachary Gruss, Alexandra Mills, Richard Lueking, Krutika Mediwala Hornback

**Affiliations:** 1 Department of Clinical Pharmacy and Outcomes Sciences, Medical University of South Carolina, Charleston, USA; 2 Department of Pharmacy Services, Medical University of South Carolina, Charleston, USA; 3 Department of Pharmacy, Cape Fear Valley Health, Fayetteville, USA; 4 Department of Pediatrics, Division of Infectious Diseases, Medical University of South Carolina, Charleston, USA; 5 Department of Internal Medicine, Division of Infectious Diseases, Medical University of South Carolina, Charleston, USA

## Abstract

**Background:**

Antimicrobial stewardship programs (ASPs) promote optimal antimicrobial use in pediatrics; however, data describing structure, personnel effort allocation, and activity distribution across regular and after-hours workflows remain limited.

**Methods:**

We conducted a cross-sectional survey of pediatric ASPs across the U.S. from September to October 2024 using ASP/ID-related listservs. Respondents reported institutional structure, personnel effort allocation, initiatives, and participation in an after-hours ASP on-call model, with additional details from programs reporting on-call participation.

**Results:**

Twenty-two pediatric ASP responses were included, most from academic medical centers (81.8%) and programs covering 101–250 beds (41%). Median physician full-time equivalents (FTEs) increased with institutional size, ranging from 0.1 for institutions with < 100 beds to 0.8 for those with >500 beds. Pharmacist FTEs were a median of 1 for institutions with ≤ 500 beds and increased to 2.25 for programs >500 beds. Most programs reported to Quality and Safety (45%) or discipline-specific departments (41%), with funding primarily attributed to Pharmacy (73%). During regular working hours, nearly all programs performed prospective audit and feedback (100%), responded to ASP-related inquiries (95.5%), and facilitated antimicrobial de-escalation (90.9%). Six programs (27.4%) reported participation in an ASP on-call model, most commonly providing remote coverage during evenings, weekends, and holidays. After-hours activities were largely limited to time-sensitive interventions, including preauthorization and responding to inquiries.

**Conclusions:**

Pediatric ASPs demonstrate variability in structure, personnel effort allocation, and stewardship activities. ASP on-call coverage remains uncommon and is typically focused on time-sensitive interventions, reflecting targeted deployment of stewardship resources outside regular working hours.

## Introduction

Antimicrobial stewardship programs (ASPs) are essential for optimizing antimicrobial use, improving patient outcomes, and reducing adverse events.^1,2^ Core elements and priority interventions have been outlined by national organizations including the Centers for Disease Control and Prevention (CDC) and the Infectious Diseases Society of America (IDSA).^2–4^ In addition, ASP implementation is required by accrediting and regulatory bodies such as Joint Commission and the Centers for Medicare and Medicaid Service.^5–7^ However, despite these frameworks and mandates for ASP implementation, there is no consensus guidance defining optimal ASP staffing models, personnel effort allocation, or “ideal” program structure, and existing requirements do not specify how much protected effort is needed to support stewardship activities.^5–7^ Furthermore, current guidance rarely differentiates between pediatric and adult patient populations, potentially overlooking pediatric-specific clinical and operational needs.

Prior studies have demonstrated substantial variability in ASP staffing models and associated effort allocation across institutions.^8–11^ In the United States (U.S.), reported full-time equivalents (FTEs) range for ASP physicians and pharmacists vary widely, with most effort dedicated to preauthorization (PRA) and prospective audit and feedback (PAF) activities.^8,9^ International reports describe higher FTE allocations for similar initiatives, though these findings vary by institution size and local standards.^10,11^ Pediatric ASPs have increased in prevalence, and most report staffing structures similar to adult programs, with high engagement in core stewardship activities.^12,13^ While these studies describe which stewardship activities are performed and which personnel are involved, they infrequently address how responsibility for these activities is distributed across time, including which team members provide coverage during and outside of regular hours, particularly in pediatric settings.

ASP activities are most commonly performed during traditional weekday hours; however, certain interventions- such as PRA for restricted antimicrobials, escalation of therapy, and questions to ASP- may be required during evenings, weekends, or holidays.^1–4^ Limited evaluations of expanded or weekend ASP services, including PAF and sterile-site culture, suggest potential benefits such as reduced antimicrobial utilization, shorter time to targeted therapy, decreased length of stay, and improved clinical outcomes.^14–19^ Despite these potential advantages, after-hours ASP coverage- particularly within pediatric programs- remains poorly defined with respect to structure, scope, staffing, and on-call support. We conducted a national survey of ASPs to examine program, structure, staffing, effort allocation, and stewardship initiatives during regular and after-hours periods and previously reported findings from responses submitted by adult institutions.^20^ In the present study, we report a pediatric-focused analysis of survey responses from pediatric institutions.

## Methods

### Study design and setting

We conducted a cross-sectional, investigator-developed survey to assess institutional structure, effort allocation, stewardship initiatives, and participation in after-hours ASP on-call model among U.S. institutions from September to October 2024. For this study, an on-call ASP model was defined as ASP-delivered stewardship activities performed outside of traditional weekday work hours (ie, Monday through Friday, 08:30 to 17:00), including weekday evenings, weekends, and major holidays.

The survey was distributed via email to the following listservs: American College of Clinical Pharmacy (ACCP) Infectious Diseases (ID) Practice and Research Network (PRN), Infectious Diseases Society of America (IDSA), Infectious Diseases Educator Network, Pediatric Infectious Diseases Society (PIDS), and Vizient Antimicrobial Stewardship. One reminder email was sent midway through the survey period. Respondents were instructed to submit one response per institution. When multiple responses were received from the same institution, the response with the highest proportion of completed survey items was retained for analysis. For the present analysis, responses indicating the institution type as a pediatric academic medical center or pediatric community hospital were included.

### Survey development and content

Survey items were developed by a multidisciplinary group of ID physicians and antimicrobial stewardship pharmacists and adapted from an informal ACCP ID PRN survey on ASP on-call models. The survey underwent iterative internal review and pilot testing among study investigators to assess clarity, feasibility, and relevance prior to distribution. The intended audience included physicians, pharmacists, or other individuals with direct involvement in ASP activities at their institution. The final instrument included 63 potential questions across 3 domains (Supplementary Materials):

1. **Institutional characteristics** (geographic location, institution type, approximate bed size)
2. **Structural characteristics** (personnel composition, FTE allocation, funding source, reporting hierarchy, and inclusion of ambulatory or outpatient services)
3. **Stewardship initiatives** (e.g., PRA, PAF, sterile-site culture review; communication and documentation practices)

The exact timing of transitions to after-hours coverage varied by institution; therefore, coverage was assessed by confirmation of ASP availability during predefined periods (weekday evenings, weekends, and major holidays), rather than specific start and end times. Institutions reporting coverage during any portion of these periods were considered to have on-call ASP coverage. Respondents indicating participation in an on-call ASP model were presented with additional questions addressing after-hours stewardship initiatives, coverage schedules, personnel involved, and compensation. Branching logic was applied based on responses to relevant questions, and free-text comment fields were available for elaboration.

The survey was created and distributed using REDCap® (Research Electronic Data Capture) hosted at the Medical University of South Carolina (MUSC).^21,22^ The MUSC Institutional Review Board reviewed the protocol and determined the study to be exempt from human subjects research prior to study commencement.

### Data analysis

Descriptive statistics were used to summarize responses. Categorical variables are reported as frequencies and percentages and continuous variables as medians with interquartile ranges (IQR). Free-text comments were abstracted and grouped by thematic similarity. Program characteristics were stratified by participation in an ASP on-call model; however, no inferential statistical analyses were performed given the exploratory nature of this study. Data were analyzed and figure generation were conducted using R (version 4.4.2 [2024]; R Foundation for Statistical Computing; Vienna, Austria).

## Results

### Survey responses and institutional characteristics

Of the 141 survey responses received, 95 were fully completed. After removal of duplicate entries, 91 unique responses were retained for review. Among these, 22 respondents represented pediatric institutions, including 18 academic medical centers (81.8%) and 4 community hospitals (18.2%). Responses were geographically diverse, with representation from all U.S. regions; the Midwest and Southeast accounted for the highest proportion of responses (14/22 [63.6%]). Most pediatric programs reported stewardship coverage of 101–250 beds (9/22 [41%]), and six programs (27.3%) reported participation in an after-hours, on-call model.

### ASP structure, leadership, and effort allocation

Nearly all pediatric ASPs reported participation by both physicians and pharmacists, with variable involvement of infection preventionists, clinical microbiologists, and information analysts. All programs identified physician leadership; however, approximately one-quarter lacked formal pharmacy leadership. Most programs directly reported to Quality and Safety programs or discipline-specific departments. Funding was most frequently attributed to the Pharmacy department. Ambulatory or outpatient stewardship presence was reported by 5 institutions (22.8%). Institutional demographics and program structural characteristics, overall and stratified by on-call participation, are summarized in Table 1.

**Table 1.** Institutional demographics and structural characteristics of pediatric antimicrobial stewardship programs Table 1 long description.

	Responses, no. (%)		
Characteristics	With On-call (n = 6)	Without On-call (n = 16)	Overall (n = 22)
Geographical location			
Northeast	0 (0%)	2 (12.5%)	2 (9.1%)
Southeast	4 (67%)	2 (12.5%)	6 (27.4%)
Midwest	1 (16.5%)	7 (44%)	8 (36.2%)
Southwest	1 (16.5%)	1 (6%)	2 (9.1%)
West	0 (0%)	4 (25%)	4 (18.2%)
Institution type			
Community hospital	1 (16.5%)	3 (19%)	4 (18.2%)
Academic medical center	5 (83.5%)	13 (81%)	18 (81.8%)
Institution size			
<100	1 (16.5%)	2 (12.5%)	3 (13.6%)
101–250	4 (67%)	5 (31%)	9 (41%)
251–500	1 (16.5%)	7 (44%)	8 (36.3%)
>500	0 (0%)	2 (12.5%)	2 (9.1%)
ASP organization			
Systemwide, with flagship hospital	2 (33.3%)	6 (37%)	8 (36.2%)
Systemwide, hospitals act independently	2 (33.3%)	5 (31.5%)	7 (31.5%)
Single facility	2 (33.3%)	5 (31.5%)	7 (31.5%)
Participating members*			
Physician(s)	6 (100%)	15 (94%)	21 (95.5%)
Pharmacist(s)	6 (100%)	16 (100%)	22 (100%)
Clinical microbiologist(s)	3 (50%)	5 (31.5%)	8 (36.2%)
Information analyst(s)	1 (16.5%)	3 (19%)	4 (18.2%)
Infection preventionist(s)	3 (50%)	6 (37%)	9 (41%)
Hospital epidemiologist(s)	1 (16.5%)	1 (6%)	2 (9.1%)
Physician leadership for ASP			
No physician leadership	0 (0%)	0 (0%)	0 (0%)
One physician leader	6 (100%)	9 (56%)	15 (68.5%)
Multiple physician leaders	0 (0%)	7 (44%)	7 (31.5%)
Pharmacist leadership for ASP			
No pharmacist leadership	0 (0%)	5 (31.5%)	5 (22.8%)
One pharmacist leader	4 (66.6%)	9 (56%)	13 (59%)
Multiple pharmacist leaders	2 (33.3%)	2 (12.5%)	4 (18.2%)
ASP reporting department(s)*			
Quality and safety	3 (50%)	7 (44%)	10 (45%)
Medicine	0 (0%)	0 (0%)	0 (0%)
Pharmacy	1 (16.5%)	2 (12.5%)	3 (14%)
Report to respective department	2 (33.3%)	7 (44%)	9 (41%)
Funding source(s)*			
Quality and safety	3 (50%)	6 (37%)	9 (41%)
Medicine	4 (66.6%)	7 (44%)	11 (50%)
Pharmacy	5 (83.5%)	11 (69%)	16 (73%)
Presence of ambulatory or outpatient ASP	2 (33.3%)	3 (19%)	5 (22.8%)
Presence of OPAT/COpAT program	3 (50%)	3 (19%)	6 (27.3%)

Abbreviations: ASP, antimicrobial stewardship program; COpAT, complex outpatient antimicrobial therapy; OPAT, outpatient parenteral antimicrobial therapy.

* Respondents could have selected more than one category.

**Figure 1.**
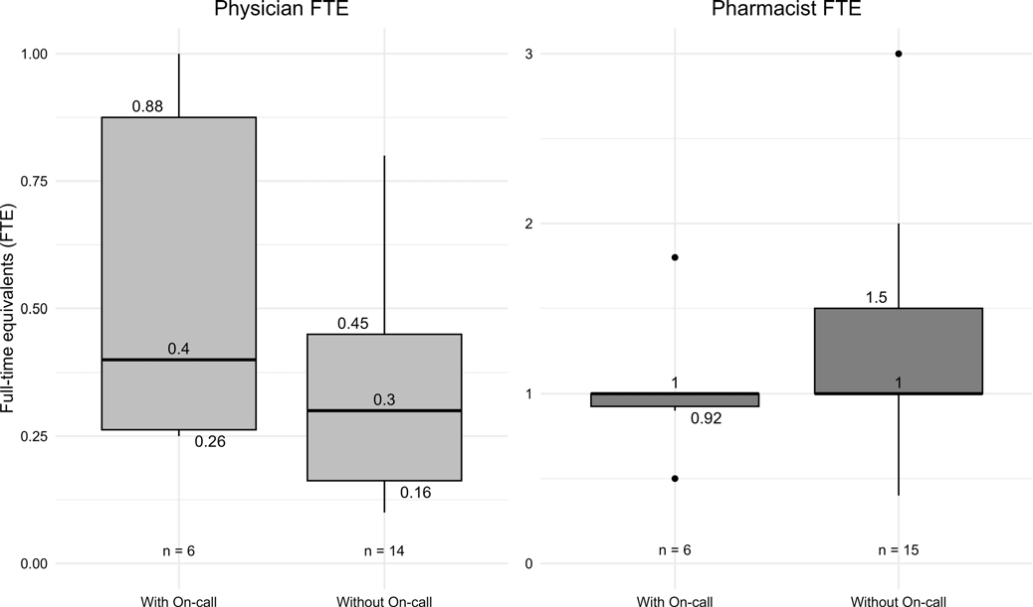
Figure 1 long description. Full-time equivalents of physicians and pharmacists with allocated effort for pediatric antimicrobial stewardship.

Effort allocation was primarily directed toward inpatient stewardship activities performed by physicians and pharmacists. Physician FTEs increased with institutional size: median FTEs (IQR) were 0.1 (0.05–0.175) for institutions with fewer than 100 beds, 0.3 (0.25–0.5) for 101–250 beds, 0.3 (0.2375–0.5) for 251–500 beds, and 0.8 (0.8–0.8) for institutions with more than 500 beds. Pharmacist FTEs demonstrated a similar pattern, with medians (IQR) of 1 (0.5–1.4), 1 (0.9–1), 1 (1–1.525), and 2.25 (1.875–2.625), respectively. FTEs stratified by ASP on-call participation are illustrated in Figure 1.

Most programs reported pharmacist support for the ID consult services, either through direct rounding (14/22 [63.6%]) or asynchronously clinical support (4/22 [18.2%]). All programs lacking pharmacy involvement in consult services were among those not participating in an on-call ASP model (4/16 [25%]). Outpatient stewardship effort was uncommon, with only three programs reporting physician FTEs and one reporting pharmacist FTEs allocated to these activities.

### Stewardship initiatives during regular working hours

**Figure 2.**
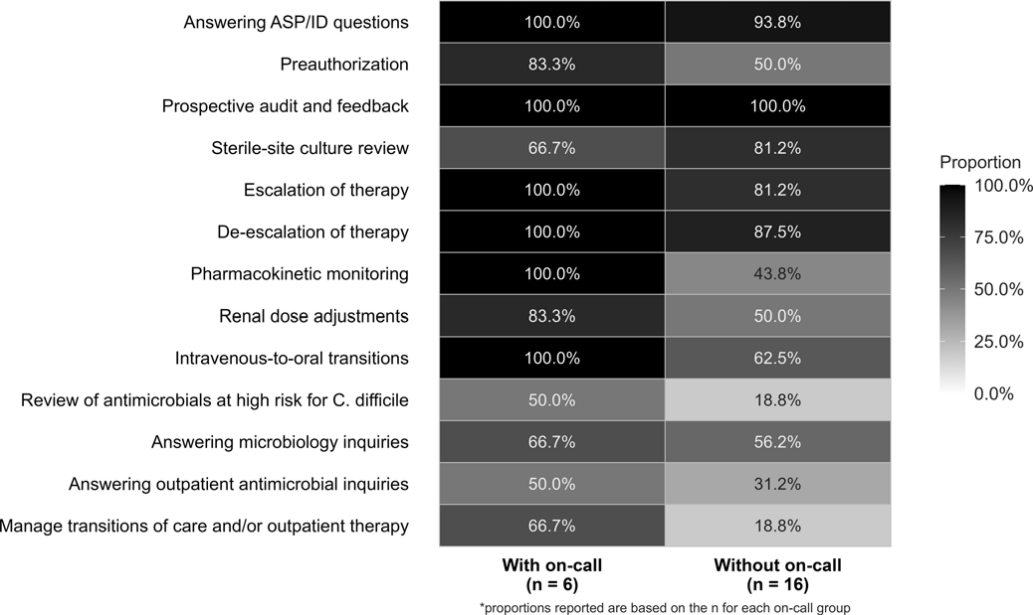
Figure 2 long description. Proportion of respondents performing select ASP initiatives based on on-call participation during regular hours.

Stewardship initiatives conducted during regular working hours, stratified by on-call participation, are shown in Figure 2. Across all pediatric programs, the most reported regular-hours activities included PAF (22/22 [100%]), responding to ASP-related inquiries (21/22 [95.5%]), and antimicrobial de-escalation (20/22 [91%]). Programs participating in on-call models more frequently reported additional initiatives such as PRA, pharmacokinetic monitoring, and intravenous-to-oral transitions compared to those without on-call ASP coverage.

Documentation practices varied across institutions. In-chart documentation was reported by 9/22 (41%) programs, while 15/22 (68.5%) used background documentation methods such as electronic intervention tracking tools (eg, i-Vents). Communication pathways to reach ASP personnel included electronic health record-integrated secure messaging (10/22 [45.5%]) and asynchronous messaging tools (3/22 [13.6%]), with additional use of Microsoft Teams (6/22 [27.4%]) and third-party secure messaging platforms (6/22 [27.4%]).

### On-call ASP coverage: organization, staffing, and operations

Six pediatric ASPs (27.3%) reported implementation of an on-call ASP model. Among these programs, five reported broad availability encompassing weekday overnight hours, weekend daytime and overnight hours, and major holidays. The sixth program reported on-call ASP availability for weekend daytime hours, but not for overnights or major holidays. Most on-call ASP services were provided remotely, with one program reporting the option for either remote or on-site coverage.

Staffing models for on-call ASP coverage varied by discipline. Physician participation in coverage was reported by four programs, all of which assigned physicians to more than 20 on-call shifts per year. Pharmacist participation was reported by three programs; two reported more than 20 shifts per year, while one reported 5–10 shifts per year. Two programs included ID fellows into their models, with fellows assigned 15–20 or more than 20 shifts annually.

**Figure 3.**
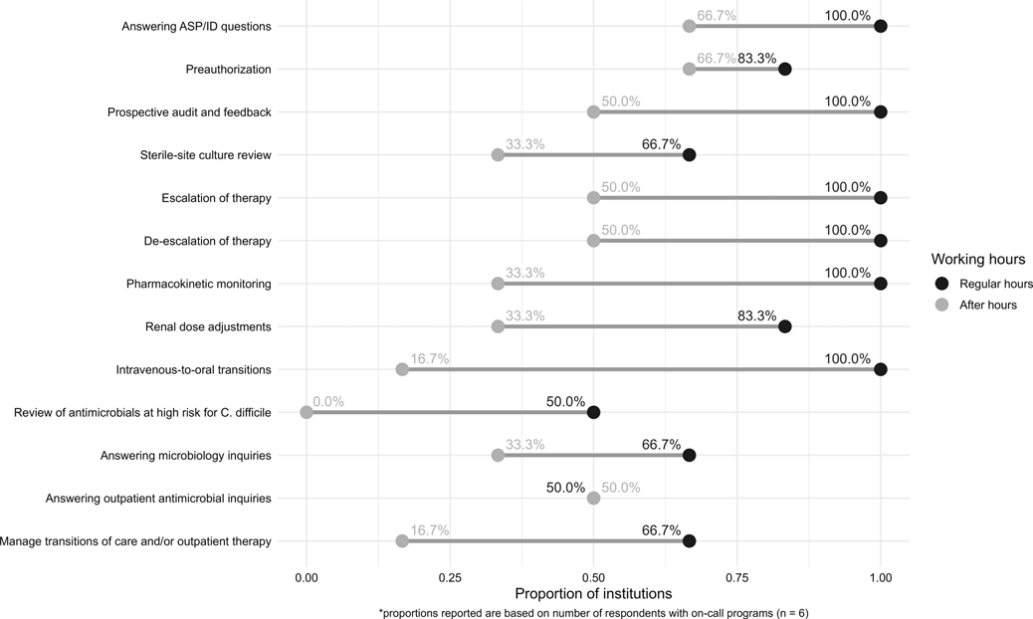
Figure 3 long description. Proportion of respondents with an on-call ASP model performing select initiatives based on working hours.

The scope of stewardship activities performed during after-hours periods was more limited compared with regular working hours. While programs continued to respond to ASP-related inquiries and perform PRA for restricted antimicrobials, fewer programs reported conducting PAF, sterile-site culture review, or intravenous-to-oral transitions during on-call coverage (Figure 3). Compensation for after-hours ASP staffing was uncommon; only one program reported the provision of incentive pay for on-call staffing. Other forms of compensation such as postcall days or paid time-off banking were not reported.

## Discussion

This survey delineates the structure, personnel effort, and initiative portfolio of pediatric ASPs in the U.S., overall and stratified by participation in on-call ASP coverage. While prior reports have characterized pediatric ASP prevalence, structure, and adherence to core elements, they have not examined how ASP activities are operationalized across working hours. These findings complement earlier work by the Sharing Antimicrobial Reports for Pediatric Stewardship (SHARPS) collaborative, which documented progress in pediatric ASP implementation but highlighted persistent gaps in resources and staffing.^12,13^

Consistent with SHARPS reports, most programs in our survey represented academic medical centers and demonstrated strong adherence to core stewardship activities during regular hours, including PAF and PRA.^12,13^ Reported median inpatient FTEs for physicians and pharmacists aligned closely with earlier studies (approximately 0.3 for physicians and 1 for pharmacists), suggesting that overall effort allocation to ASP has remained relatively stable despite regulatory mandates for stewardship activities.^5–7^ Notably, pharmacist FTEs in our survey remained largely stable across institution sizes until exceeding 500 beds, differing from the incremental increases by covered bed number reported in the cross-sectional analysis by Doernberg’s et al.^9^ This pattern may reflect a minimum staffing threshold required to sustain core stewardship activities in pediatric settings rather than proportional scaling with hospital size. ASP leadership was generally inclusive of both physicians and pharmacists; however, approximately one-quarter reported lacking formal pharmacy leadership. As previously described, outpatient stewardship resources were limited, with only 3 of 22 programs reporting physician or pharmacist effort allocation. Allocation of effort to other specialists, including clinical microbiologists and information analysts, was uncommon in our survey, contrasting with prior reports where most programs reported some degree of data analyst support.^12,13^

Stratification by on-call participation revealed that only a minority of pediatric ASPs reported dedicated after-hours coverage. Programs with an on-call ASP model reported a broader range of stewardship initiatives compared to programs without on-call coverage; however, this was not accompanied by substantial differences in physicians or pharmacists FTEs. Among programs with on-call coverage, after-hours ASP activities were typically delivered remotely and focused on time-sensitive interventions, such as PRA and responding to ASP-related inquiries, compared with initiatives performed during regular working hours. This pattern likely reflects the need to prioritize interventions with the greatest potential for immediate clinical impact when staffing and resources are constrained. Conversely, limited adoption of an on-call ASP model may reflect competing responsibilities and finite staffing rather than lack of perceived value. In this context, some researchers have suggested that stewardship impact may be enhanced by shifting greater ownership of antimicrobial decision-making to frontline clinicians when ASP resources are limited.^23^

Like our previous report of ASPs in adult institutions, participation in an on-call ASP model was reported by a minority of programs (35% adults vs 27% pediatrics), underscoring that after-hours stewardship remains non-universal across care settings. In the adult cohort, programs with on-call ASP coverage reported higher median inpatient physician (0.5 vs 0.25) and pharmacist (2.9 vs 1.45) FTE allocations compared with those without on-call coverage, whereas pediatric programs did not exhibit meaningful differences in FTE allocation based on on-call participation. From a structural standpoint, adult programs with an on-call ASP model more frequently reported pharmacist participation in after-hours coverage (62.5% reporting at least one on-call shift per year) and were more commonly based at larger institutions (75% with >500 beds), whereas pediatric programs with on-call coverage were more variably staffed, potentially reflecting differences in patient volume and overlap with ID consult services. Additionally, adult ASPs with on-call models maintained a broader portfolio of after-hours stewardship activities compared with pediatric programs.^20^ Together, these contrasts suggest that pediatric ASPs may rely more heavily on targeted, high-yield after-hours interventions rather than workforce expansion to meet stewardship needs outside traditional working hours.

Several limitations should be considered when interpreting these findings. First, the survey was distributed electronically via ASP/ID-related listservs to maximize outreach; however, this approach precluded calculation of a response rate and limited assessment of non-responder characteristics. As a result, selection bias is possible, as programs with greater stewardship infrastructure, academic affiliation, or interest in on-call ASP models may have been more likely to respond. Second, despite inclusion of international listservs, responses were exclusively from U.S.-based institutions, constraining our findings to U.S. healthcare finance models. Third, characteristics derived from free-text “other” responses may have been misclassified despite careful review of free-text comments. Fourth, the survey was not designed to determine whether physician-provided after-hours stewardship was delivered through a dedicated ASP on-call service distinct from ID consultation responsibilities, which may differ in workflow, reimbursement mechanisms, and availability of professional fee billing, thereby limiting attribution to discrete ASP on-call models. Finally, the survey did not capture the intensity or scope of specific initiatives (eg, PAF for all antimicrobials vs selected high-risk agents), the precise timing of after-hours activities, or perceived workload and burnout associated with on-call coverage, all of which may influence feasibility and sustainability of on-call ASP models.

This survey characterizes the structure, personnel effort, and stewardship activities of pediatric ASPs in the U.S., with particular emphasis on the presence and organization of on-call ASP coverage. Most responding programs represented academic medical centers, demonstrated strong adherence to core stewardship activities during regular working hours, and reported physician and pharmacist effort allocations consistent with prior national assessments. Only a minority of pediatric ASPs reported participation in an on-call model. When present, on-call coverage was most often delivered remotely and focused on time-sensitive interventions, suggesting a targeted deployment of ASP resources outside routine hours. Together, these findings underscore heterogeneity in how pediatric ASPs operationalize stewardship across time and highlight the potential influence of staffing and resource constraints on after-hours service provision. Future studies should examine the clinical, operational, and workforce implications of different stewardship coverage strategies and identified resource models that support sustainable pediatric ASP engagement across diverse care settings.

## Supporting information

10.1017/ash.2026.10745.sm001 Alvira-Arill et al. supplementary material Alvira-Arill et al. supplementary material

## Supplementary material

The supplementary material for this article can be found at https://doi.org/10.1017/ash.2026.10745

## Table 1. Long description

The table presents institutional demographics and structural characteristics of pediatric antimicrobial stewardship programs, comparing those with and without on-call participation. It includes data on geographical location, institution type, institution size, ASP organization, participating members, physician and pharmacist leadership for ASP, ASP reporting department, funding sources, and the presence of ambulatory or outpatient ASP and OPAT/COPAT programs. The table has 18 rows and 7 columns, with column headers including Characteristics, With On-call, Without On-call, and Overall. Notable trends include a higher concentration of academic medical centers, significant involvement of physicians and pharmacists, and varied leadership structures. Most programs report to Quality and Safety or discipline-specific departments, with funding primarily from the Pharmacy department.

Navigate back to Table 1..

## Figure 1. Long description

A box-and-whisker plot compares the full-time equivalents of physicians and pharmacists with and without on-call duties. The plot consists of two vertical box plots for physicians and two for pharmacists. The x-axis represents the categories ‘With On-call’ and ‘Without On-call’ for both physicians and pharmacists, while the y-axis represents full-time equivalents in terms of FTE. For physicians with on-call duties, the median is 0.4 FTE, with a lower quartile of 0.26 FTE and an upper quartile of 0.88 FTE. The range extends from 0.26 to 0.88 FTE, with no outliers. For physicians without on-call duties, the median is 0.3 FTE, with a lower quartile of 0.16 FTE and an upper quartile of 0.45 FTE. The range extends from 0.16 to 0.45 FTE, with no outliers. For pharmacists with on-call duties, the median is 1 FTE, with a lower quartile of 0.92 FTE and an upper quartile of 1 FTE. The range extends from 0.92 to 1 FTE, with one outlier above 3 FTE. For pharmacists without on-call duties, the median is 1 FTE, with a lower quartile of 1 FTE and an upper quartile of 1.5 FTE. The range extends from 1 to 1.5 FTE, with no outliers. The dataset includes 6 observations for physicians with on-call duties, 14 for physicians without on-call duties, 6 for pharmacists with on-call duties, and 15 for pharmacists without on-call duties. All values are approximated.

Navigate back to Figure 1..

## Figure 2. Long description

The bar graph compares the proportion of respondents performing select antimicrobial stewardship program initiatives based on on-call participation during regular hours. The x-axis lists various initiatives such as answering A S P slash I D questions, preauthorization, prospective audit and feedback, sterile-site culture review, escalation of therapy, de-escalation of therapy, pharmacokinetic monitoring, renal dose adjustments, intravenous-to-oral transitions, review of antimicrobials at high risk for C. difficile, answering microbiology inquiries, answering outpatient antimicrobial inquiries, and managing transitions of care and or outpatient therapy. The y-axis represents the proportion in percentage. There are two sets of horizontal bars: one for respondents with on-call participation and one for those without. The bars are color-coded to indicate the proportion, with darker shades representing higher proportions. For example, the initiative ‘Answering A S P slash I D questions’ shows a proportion of one hundred percentage for respondents with on-call participation and ninety-three point eight percentage for those without. The graph highlights variations in the implementation of different initiatives based on on-call participation. All values are approximated.

Navigate back to Figure 2..

## Figure 3. Long description

A line graph illustrates the proportion of institutions performing various antimicrobial stewardship activities during regular and after hours. The x-axis represents the proportion of institutions, ranging from 0.00 to 1.00. The y-axis lists different activities such as answering ASP/ID questions, preauthorization, prospective audit and feedback, sterile-site culture review, escalation of therapy, de-escalation of therapy, pharmacokinetic monitoring, renal dose adjustments, intravenous-to-oral transitions, review of antimicrobials at high risk for C. difficile, answering microbiology inquiries, answering outpatient antimicrobial inquiries, and managing transitions of care and/or outpatient therapy. Each activity is represented by two data points: one for regular hours and one for after hours. For example, answering ASP/ID questions is performed by 100 percentage of institutions during regular hours and 66.7 percentage during after hours. Prospective audit and feedback is performed by 100 percentage during regular hours and 50 percentage during after hours. All values are approximated.

Navigate back to Figure 3..
