# Supplementary material for: Structure, personnel, and initiatives of antimicrobial stewardship programs in pediatric hospitals in the United States based on on-call participation: a cross-sectional survey
Source: Antimicrob Steward Healthc Epidemiol. 2026 Jun 10;6(1):e173. doi: 10.1017/ash.2026.10745 (PMC13273151; doi:10.1017/ash.2026.10745)
Supplement: Alvira-Arill et al. supplementary material [file S2732494X26107451sup001.pdf]

Antimicrobial Stewardship Program On-Call Survey

The Medical University of South Carolina (MUSC) Health is conducting a web-based survey to characterize the practices of antimicrobial stewardship program (ASP) On-Call models and practices among institutions across the United States.

You are being contacted because your institution may currently have or participate in an ASP On-Call program. Your participation in this survey is voluntary and will not collect personal information. You may refuse to take part in the research or exit the survey at any time without penalty. You are free to decline to answer any particular question you do not wish to answer for any reason.

This study has received IRB approval from MUSC (The MUSC Exempt Research Protocol Number is Pro00139136). There is a risk of loss of confidentiality with participation. However, the survey does not collect any identifying information. No one will be able to identify you or your answers, and no one will know whether or not you participated in the study.

This survey should take about 15 minutes to complete. Completing the survey implies your understanding of research procedures and your willingness to participate. You can return to the survey as needed until you click "submit".

Thank you for your consideration and we look forward to your survey responses. For any questions related to the survey email from our site, please contact:

Samantha Brace, PharmD; bricesa@musc.edu

Krutika Mediwala Hornback, PharmD; mediwala@musc.edu

General Demographics

What is the name of your institution? (only requested to remove potential, duplicate responses)	__________________________________
Where is your institution located? (reference: https://education.nationalgeographic.org/resource/united-states-regions/)	◯ Midwest<br>◯ Northeast<br>◯ Southeast<br>◯ Southwest<br>◯ West
Which of the following best describes your institution?	◯ Academic medical center<br>◯ Community hospital<br>◯ Critical access hospital<br>◯ Specialty hospital<br>◯ Veteran Affairs or Federal hospital<br>◯ Pediatric community hospital<br>◯ Pediatric academic medical center<br>◯ Rehabilitation hospital<br>◯ Long-term acute care facilities
What is the approximate number of beds covered by your ASP program? (if system-wide, please include total count)	◯ < 100<br>◯ 101 - 250<br>◯ 251 - 500<br>◯ > 500

ASP Background and Information

Select the description that best matches your ASP program to the number of institutions covered:	◯ System-wide ASP services, with one flagship hospital<br>◯ System-wide ASP services, with each hospital having independent ASP programs<br>◯ Single-site ASP services
Do you have an established ambulatory/outpatient ASP program?	◯ Yes<br>◯ No
Do you have an established OPAT/COpAT program?	◯ Yes<br>◯ No
Which department does the ASP program report to?	◯ Quality and Safety<br>◯ Medicine<br>◯ Pharmacy<br>◯ Members of each discipline reports to their respective department (e.g., physicians report to Medicine, pharmacists report to Pharmacy)<br>◯ Other
Please specify "other"	__________________________________
Who is responsible for the majority funding of the ASP program? (select all that apply)	☐ Quality and Safety<br>☐ Medicine<br>☐ Pharmacy<br>☐ Other
Please specify "other"	__________________________________
Which of the following are included in the ASP team? (select all that apply)	☐ Physician(s) (MD/DOs)<br>☐ Pharmacist(s)<br>☐ Clinical microbiologist(s)<br>☐ Information system specialist(s)<br>☐ Infection prevention personnel<br>☐ Hospital epidemiologist(s)<br>☐ Other (e.g., advanced practice provider [APP], statistician, etc.)
Which areas are covered through the dedicated physician FTE for your ASP program?	☐ Inpatient<br>☐ Outpatient
Physician inpatient FTE	__________________________________
Physician outpatient FTE	__________________________________
Which areas are covered through the dedicated pharmacist FTE for your ASP program?	☐ Inpatient<br>☐ Outpatient
Pharmacist inpatient FTE	__________________________________

Pharmacist outpatient FTE	__________________________
Which areas are covered through the dedicated clinical microbiologist(s) FTE for your ASP program?	☐ Inpatient<br>☐ Outpatient
Clinical microbiologist(s) inpatient FTE	__________________________
Clinical microbiologist(s) outpatient FTE	__________________________
Which areas are covered through the dedicated information system specialist(s) FTE for your ASP program?	☐ Inpatient<br>☐ Outpatient
Information system specialist(s) inpatient FTE	__________________________
Information system specialist(s) outpatient FTE	__________________________
Which areas are covered through the dedicated infection prevention personnel FTE for your ASP program?	☐ Inpatient<br>☐ Outpatient
Infection prevention personnel inpatient FTE	__________________________
Infection prevention personnel outpatient FTE	__________________________
Which areas are covered through the dedicated hospital epidemiologist(s) FTE for your ASP program?	☐ Inpatient<br>☐ Outpatient
Hospital epidemiologist(s) inpatient FTE	__________________________
Hospital epidemiologist(s) outpatient FTE	__________________________
Please specify "other"	__________________________
Which areas are covered through the dedicated "other" FTE for your ASP program?	☐ Inpatient<br>☐ Outpatient
Other inpatient FTE	__________________________
Other outpatient FTE	__________________________
Does included ASP pharmacist FTE also provide support for the ID consult service?	○ Yes<br>○ No

How do ASP pharmacists support the ID consult service?	◯ Consistent rounding (either in-person OR virtual)<br>◯ Available to answer questions
Who are the recognized leaders of the ASP Program? (i.e., hold leadership title(s) and have leadership incorporated into job description [select all that apply])	☐ One physician<br>☐ Multiple physicians (shared responsibilities)<br>☐ One pharmacist<br>☐ Multiple pharmacists (shared responsibilities)<br>☐ Other
Please specify "other"	__________________________________
Is the ASP program able to bill for its consult services?	◯ Yes<br>◯ No
If "yes," please explain how the ASP program is able to bill for consultation	__________________________________

ASP Initiatives and On-call

What initiatives are performed during daytime hours on ASP staffing? (select all that apply)

- ☐ Answering general ADULT ASP/ID questions
- ☐ Answering general PEDIATRIC ASP/ID questions
- ☐ Preauthorization (i.e., restricted antimicrobial review and approval)
- ☐ Prospective audit and feedback of protected antimicrobials (e.g., time-outs)
- ☐ Sterile-site prospective audit and feedback (e.g., rapid diagnostics, blood cultures, resistance alerts)
- ☐ Escalation of therapy (e.g., patient with positive MRSA blood culture on no therapy)
- ☐ De-escalation of therapy (e.g., piperacillin-tazobactam to ceftriaxone for susceptible E. coli)
- ☐ Pharmacokinetic monitoring (e.g., vancomycin, aminoglycosides)
- ☐ Renal dose adjustments
- ☐ IV-to-PO conversions
- ☐ Review of agents posing high risk for C. difficile infection (CDI)
- ☐ Answer general microbiology questions
- ☐ Answering general OUTPATIENT antimicrobial questions
- ☐ Manage transitions of care and/or outpatient parenteral antibiotic therapy (OPAT)
- ☐ Other

Please specify "other"

____

What documentation/handoff is performed for ASP interventions? (select all that apply)

- ☐ In-chart documentation (e.g., progress notes, consult notes, etc.)
- ☐ Background documentation in EMR (e.g., Epic Stewardship Navigator, i-vents)
- ☐ Third party software (e.g., Theradoc, Vigilanz)
- ☐ No documentation performed
- ☐ Other

Please specify "other"

____

What forms of communication are used to reach ASP? (select all that apply)

- ☐ Dedicated telephone line
- ☐ Dedicated pager
- ☐ Secure chat communication in EMR
- ☐ In-basket messages in EMR
- ☐ Microsoft Teams
- ☐ Email
- ☐ Other

Please specify "other"

____

Do you have an after-hours (e.g., 5:00 PM - 8:00 AM, weekends, holidays), on-call ASP program?

- ◯ Yes
- ◯ No

What times is the after-hours, on-call ASP program available? (select all that apply)	☐ Weekdays after work hours (e.g., 4:00 PM - 7:00 AM, 5:00 PM - 8:00 AM, etc.)<br>☐ Weekends daytime hours (e.g., 7:00 AM - 4:00 PM, 8:00 AM - 5:00 PM, etc.)<br>☐ Weekends after work hours (e.g., 4:00 PM - 7:00 AM, 5:00 PM - 8:00 AM, etc.)<br>☐ Major holidays (i.e., Christmas, Independence Day, Labor Day, Memorial Day, New Year's Day, and Thanksgiving)
What location is the after-hours, on-call ASP program staffed from? (select all that apply)	☐ Remote, at-home<br>☐ Remote, designated off-hospital campus site<br>☐ In-person, on-site at hospital
What initiatives are performed during after-hours, on-call ASP staffing? (select all that apply)	☐ Answering general ADULT ASP/ID questions<br>☐ Answering general PEDIATRIC ASP/ID questions<br>☐ Preauthorization (i.e., restricted antimicrobial review and approval)<br>☐ Prospective audit and feedback of protected antimicrobials (e.g., time-outs)<br>☐ Sterile-site prospective audit and feedback (e.g., rapid diagnostics, blood cultures, resistance alerts)<br>☐ Escalation of therapy (e.g., patient with positive MRSA blood culture on no therapy)<br>☐ De-escalation of therapy (e.g., piperacillin-tazobactam to ceftriaxone for susceptible E. coli)<br>☐ Pharmacokinetic monitoring (e.g., vancomycin, aminoglycosides)<br>☐ Renal dose adjustments<br>☐ IV-to-PO conversions<br>☐ Review of agents posing high risk for C. difficile infection (CDI)<br>☐ Answer general microbiology questions<br>☐ Answering general OUTPATIENT antimicrobial questions<br>☐ Manage transitions of care and/or outpatient parenteral antibiotic therapy (OPAT)<br>☐ Other
Please specify "other"	______________________________
Who participates in the ASP after-hours, on-call program? (select all that apply)	☐ ASP Physician(s)<br>☐ ASP Pharmacist(s)<br>☐ ID Medical Fellow(s)<br>☐ PGY-2 ID Pharmacy Resident(s)<br>☐ Other

How often is each ASP physician on-call during the calendar year?	◯ 1 - 5 shifts per year<br>◯ 5 - 10 shifts per year<br>◯ 10 - 15 shifts per year<br>◯ 15 - 20 shifts per year<br>◯ > 20 shifts per year<br>(Shifts are defined as any of the following: weekdays after work hours (e.g., 4:00 PM - 7:00 AM, 5:00 PM - 8:00 AM, etc.), weekends daytime hours (e.g, 7:00 AM - 4:00 PM, 8:00 AM - 5:00 PM, etc.), weekends after work hours (e.g., 4:00 PM - 7:00 AM, 5:00 PM - 8:00 AM, etc.), major holidays (i.e., Christmas, Independence Day, Labor Day, Memorial Day, New Year's Day, and Thanksgiving))
How often is each ASP pharmacist on-call during the calendar year?	◯ 1 - 5 shifts per year<br>◯ 5 - 10 shifts per year<br>◯ 10 - 15 shifts per year<br>◯ 15 - 20 shifts per year<br>◯ > 20 shifts per year<br>(Shifts are defined as any of the following: weekdays after work hours (e.g., 4:00 PM - 7:00 AM, 5:00 PM - 8:00 AM, etc.), weekends daytime hours (e.g, 7:00 AM - 4:00 PM, 8:00 AM - 5:00 PM, etc.), weekends after work hours (e.g., 4:00 PM - 7:00 AM, 5:00 PM - 8:00 AM, etc.), major holidays (i.e., Christmas, Independence Day, Labor Day, Memorial Day, New Year's Day, and Thanksgiving))
How often is each ID medical fellow on-call during the calendar year?	◯ 1 - 5 shifts per year<br>◯ 5 - 10 shifts per year<br>◯ 10 - 15 shifts per year<br>◯ 15 - 20 shifts per year<br>◯ > 20 shifts per year<br>(Shifts are defined as any of the following: weekdays after work hours (e.g., 4:00 PM - 7:00 AM, 5:00 PM - 8:00 AM, etc.), weekends daytime hours (e.g, 7:00 AM - 4:00 PM, 8:00 AM - 5:00 PM, etc.), weekends after work hours (e.g., 4:00 PM - 7:00 AM, 5:00 PM - 8:00 AM, etc.), major holidays (i.e., Christmas, Independence Day, Labor Day, Memorial Day, New Year's Day, and Thanksgiving))
How often is each PGY-2 ID pharmacy resident on-call during the calendar year?	◯ 1 - 5 shifts per year<br>◯ 5 - 10 shifts per year<br>◯ 10 - 15 shifts per year<br>◯ 15 - 20 shifts per year<br>◯ > 20 shifts per year<br>(Shifts are defined as any of the following: weekdays after work hours (e.g., 4:00 PM - 7:00 AM, 5:00 PM - 8:00 AM, etc.), weekends daytime hours (e.g, 7:00 AM - 4:00 PM, 8:00 AM - 5:00 PM, etc.), weekends after work hours (e.g., 4:00 PM - 7:00 AM, 5:00 PM - 8:00 AM, etc.), major holidays (i.e., Christmas, Independence Day, Labor Day, Memorial Day, New Year's Day, and Thanksgiving))
Please specify the "other" ASP member that participates in the on-call program	__________________________________

How often is this "other" ASP member on-call during the calendar year?	◯ 1 - 5 shifts per year<br>◯ 5 - 10 shifts per year<br>◯ 10 - 15 shifts per year<br>◯ 15 - 20 shifts per year<br>◯ > 20 shifts per year<br>(Shifts are defined as any of the following: weekdays after work hours (e.g., 4:00 PM - 7:00 AM, 5:00 PM - 8:00 AM, etc.), weekends daytime hours (e.g, 7:00 AM - 4:00 PM, 8:00 AM - 5:00 PM, etc.), weekends after work hours (e.g., 4:00 PM - 7:00 AM, 5:00 PM - 8:00 AM, etc.), major holidays (i.e., Christmas, Independence Day, Labor Day, Memorial Day, New Year's Day, and Thanksgiving))
Is there any compensation or benefits offered for staffing the after-hours, on-call ASP program (e.g., monetary, post-call day off)?	◯ Yes<br>◯ No
What form of compensation is offered?	☐ Post-call day off<br>☐ Incentive pay<br>☐ PTO banking<br>☐ Other
Please specify "other"	__________________________________
Please provide any other information regarding your ASP program that you find important and/or noteworthy:	____
